# Potential role of vacuum-assisted procedures in resecting breast cancers and highlighting selection criteria to support future trials

**DOI:** 10.3389/fonc.2023.1239574

**Published:** 2023-09-21

**Authors:** C. N. Valadares, H. L. Couto, A. N. Soares, P. H. Toppa, B. P. Ricardo, S. A. McIntosh, N. Sharma, V. Resende

**Affiliations:** ^1^ Universidade Federal de Minas Gerais, Belo Horizonte, Brazil; ^2^ Sociedade Brasileira de Mastologia, Rio de janeiro, Brazil; ^3^ Faculdade Santa Casa de Belo Horizonte, Minas Gerais, Brazil; ^4^ Patrick G Johnston Centre for Cancer Research, Queen’s University Belfast, Belfast, United Kingdom; ^5^ Breast Unit, Leeds Teaching Hospital NHS Trust, St James Hospital, Leeds, United Kingdom

**Keywords:** vacuum assisted biopsy, breast cancer, minimally invasive procedure, breast, biopsy

## Abstract

**Purpose:**

The purpose of this study was to evaluate the role of vacuum-assisted biopsy (VAB) in resecting breast cancers.

**Methods:**

Retrospective database analysis of 116 cancers [both invasive breast cancers (IC) and ductal carcinoma *in situ* (DCIS)] diagnosed by VAB submitted to standard surgical treatment with complete histological data from VAB and surgery. Excision following VAB was defined as complete resection (CR) if there was no residual tumor in the surgical specimen, minimal residual disease (MRD) if residual tumor ≤ 3 mm, gross residual disease (GRD) if residual tumor > 3 mm, and upgrade from DCIS on VAB to IC. CR and MRD were combined as potentially resected percutaneously (PRP). GRD and those with upgrade to IC were determined not eligible for percutaneous resection (NPR). Factors predictive of PRP were evaluated.

**Results:**

Mean age was 55.6 years (20–91; SD: 12,27). CR was seen in 29 of 116 cases (25%), MRD in 18 of 116 cases (15.5%), GRD in 64 of 116 cases (55.2%), and five of 116 cases (4.3%) were upgraded from DCIS to IC, and those groups combined represented 47 cases of PRP (40.5%) and 69 (59,5%) of NPR. For 77 tumors ≤ 10 mm, 45 (58.5%) were PRP. Multivariate analysis reveals significance for enlarged VAB (EVAB) (*p* = 0.008, OR: 4.4, 95% CI), low/intermediate nuclear grade (*p* < 0.001, OR: 12.5, 95% CI) and final tumor size (T) ≤ 10 mm (*p* = 0.001, OR: 50.1, 95% CI) for PRP.

**Conclusions:**

This study showed that lesions completely excised with VAB that were cancer could have been treated with VAB rather than surgery but tumor selection in terms of subtype and size is important.

## Introduction

In 2020, breast cancer was the most diagnosed female cancer, with an estimated 2.3 million new cases (11.7%) globally. It is the fifth leading cause of cancer deaths, accounting for 6.9% of cancer deaths worldwide (1). Since 1988–1995, mortality from breast cancer has been declining sharply in most high-income countries (2). The inception of mammographic screening programs has led to increasing diagnosis of small breast cancers, many of which have favorable biological characteristics (3, 4). Some of these tumors have excellent long-term outcomes, with 10-year breast cancer specific survival approaching 100% (5). It is likely that such tumors may never become symptomatic within a patient’s lifetime due to their indolent nature and may thus represent overdiagnosis. There has been much debate around the extent of overdiagnosis within mammographic screening programs. In the UK, an Independent Panel Review of the UK NHS Breast Screening Program concluded that, for every breast cancer death prevented by screening, around three cancers were overdiagnosed and consequently overtreated (6). However, despite improvements in the understanding of tumor biology, there remains no way of identifying those small (T1a and T1b) tumors with broadly favorable characteristics (e.g., hormone receptor positive [HR+] and HER2−) that are likely to progress or to become life threatening. These patients are all generally treated the same manner, usually with breast-conserving surgery, adjuvant radiotherapy, and endocrine therapy. These treatment modalities have associated physical and psychological morbidities for patients and costs for healthcare providers (7–9). Thus, there is increasing interest in the de-escalation of loco-regional therapies for small screen-detected tumors.

Numerous image-guided ablative techniques have been described for the minimally invasive management of small tumors (10). By their nature, however, majority of these techniques do not provide tumor tissue for histopathological assessment and often require specialized equipment and expertise, which is not readily available. However, vacuum-assisted excision (VAE) is a commonly used technique for both diagnostic and therapeutic purposes and can be carried out under local anesthesia and image guidance. VAE is currently used to manage benign lesions (11) and lesions of uncertain malignant potential (B3 lesions), where the aim is to take 4 g of tissue, which would remove the lesion if small (≤ 15 mm in size) but otherwise allow representative sampling without complete resection (CR) if > 15 mm in size (12–14). More recently, there has been interest in the application of VAE for the percutaneous resection and treatment of small breast cancers to potentially reduce morbidity and surgical overtreatment of screen-detected cancers (15).

This retrospective series aimed to evaluate the role of vacuum-assisted procedures in the resection of breast cancers and support selection criteria for future prospective trials.

## Methods

### Patient eligibility and study design

The study was approved by the Ethical Committee of Santa Casa de Belo Horizonte by the number 25761019.8.0000.5138, and all methods were performed in accordance with the relevant guidelines. Written informed consent was obtained from all patients for participation. The datasets used and/or analyzed during the current study available from the corresponding author on reasonable request.

A total of 1,061 vacuum-assisted biopsy (VAB) for suspicious breast lesions categorized as BI-RADS 4 and BI-RADS 5 or lesions with uncertain malignant potential in previous core biopsy (B3) performed in a single breast unit in Brazil from 13/04/2017 to 28/11/2020 were analyzed. Patients who had benign histological findings on VAB, who had malignancy confirmation but did not undergo final definitive surgery, or where final surgical pathology was unavailable were excluded. This resulted in 116 cancers (invasive and non-invasive) with complete data from surgical excision that were included in the analysis. The lesions were classified as mass, mass with calcifications or calcifications.

Baseline demographic data were recorded. Data were collected on imaging, including baseline assessments, findings (mass ± calcification), image-guided approach to VAB (US/stereotactic), and maximum radiological tumor size (TI).

### VAB procedure

A diagnostic VAB was carried out. The VAB either resulted in representative sampling or the lesion was excised in its entirety as these were diagnostic biopsies. After all the procedures, a mammogram was taken to evaluate clip marker position. The vacuum-assisted procedures were defined as ordinary VAB *versus* enlarged VAB (EVAB) when the lesion was completely excised by image or more than 12 samples with a 7G needle or 18 samples with a 10G needle were rescued. Biopsy device (EnCor Enspire™ Breast Biopsy System – BD or Mammotome Revolve™ Dual Vacuum-Assisted Breast Biopsy System) and needle gauge used were at the discretion of the operating physician.

### VAB pathological reports

Gross specimens are separated from the clots, measured, weighted, and inked. Total inclusion of the fragments is performed, and slices are cut every four microns. Cases vary, on average, from one to five blocks of paraffin. Tests range from a usual HE analysis on slides, with or without immunohistochemistry at the discretion of the case by the pathologist, as well as followed by FISH and genetic analyses (e.g., oncotype) if indicated.

All tissue samples were submitted for histopathological evaluation. Maximum pathological tumor size following VAB was defined as the measure of the maximum size of tumor in the slide of the greatest core sample compromised by tumor. Following assessment, VAB pathology diagnosis (invasive disease ± DCIS), presence of DCIS with comedonecrosis, biomarker status (ER/PR/HER2/Ki67), morphological tumor type, and nuclear and histological grades were all recorded. In case of multicentric or bilateral breast cancers, only tumor measurements and outcomes of the tumor submitted to VAB were included. One patient with two multicentric nodules was submitted to two different VAB procedures, so this case was treated as two lesions.

### Surgical procedure and pathological reports

All cases underwent surgical excision following VAB. After surgery, radiography of surgical specimen was performed to confirm the presence of the marker placed at VAB. Gross surgical specimens are measured, weighted, and inked. All surgically excised tissue was submitted for histopathological evaluation and slices are cut every four microns. Tests range from a usual HE analysis on slides, with or without immunohistochemistry at the discretion of the case by the pathologist, as well as followed by FISH and genetic analyses (e.g., oncotype) if indicated. Following assessment, maximum pathological residual tumor size, diagnosis (invasive disease ± DCIS), presence of DCIS with comedonecrosis, multifocality, biomarker status (ER/PR/HER2/Ki67), morphological tumor type, and nuclear and histological grades were all recorded. Sentinel node biopsy (SNB) was performed according to clinical practice (16).

Presence of residual invasive or *in situ* disease on the surgical specimen was noted. Excision following VAB was defined as CR if there was no residual tumor at surgery, minimal residual disease (MRD) if residual tumor ≤ 3 mm, gross residual disease (GRD) if residual tumor > 3 mm, and upgrade from DCIS on VAB to invasive cancer. In this study, we used 7G to 10G needles. A 7G (4.57 mm in diameter) needle provides a core sample that weights 0.363 g. The 3 mm cut off for MRD was defined based on the smallest single core sample that weights 0.221 g and is provided by a 10G (3.5 mm in diameter) (12). In this way, 3 mm of residual disease could be easily resected by one or two core samples.

CR and MRD were combined and considered as potentially resected percutaneously (PRP) by VAB in a supposed intent to treat procedure. GRD and tumors where an upgrade from DCIS to invasive disease was seen at excision were defined as not eligible for percutaneous resection (NPR).

### Adjuvant treatment

All patients received adjuvant systemic therapy and radiotherapy according to Brazilian´s Guideline for Breast Cancer Diagnose and Treatment from Brazilian Health Department (16).

### Statistical analysis

Categorical data were summarized as counts and relative frequencies, and comparisons between groups of interest (tumors PRP × tumors NPR) were performed using the chi-square test or Fisher’s exact test depending on each specific case.

Continuous variables were summarized as mean ± standard deviation (SD) and range. For continuous parametric variables, comparisons between groups were made using a two-sample t-test.

To identify possible imaging and pathologic characteristics of breast cancers that could predict potential CR, univariate and multivariate analyses were performed. For multivariate analyses, variables with at least 80% of total number of observations and *p* ≤ 0.20 were included. Odds Ratio (OR) was calculated by the “Backward Model.” In the final logistic multivariate model, variables with statistical significance of *p* < 0.05 were retained. For definition of the final models the test likelihood ratio test was used. Analyses were carried out using SPSS 20.0 (IBM SPSS Version 20.0, Armonk, NY).

## Results

**Figure 1:**
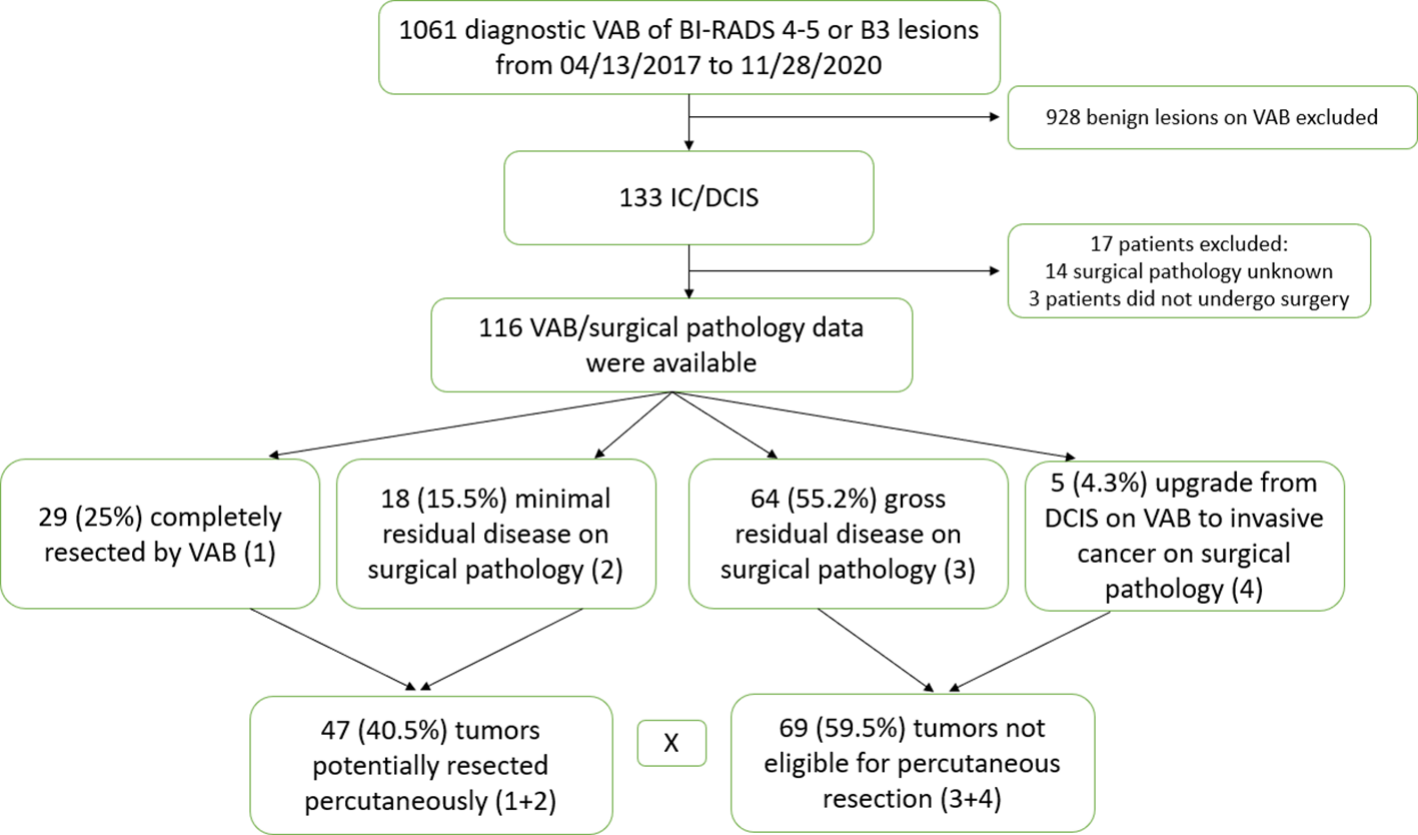
Patient identification.

One thousand sixty-one diagnostic VAB procedures were reviewed. One hundred thirty-three breast cancers were identified. Seventeen cases were excluded due to lack of pathological data following subsequent surgical excision. One hundred sixteen lesions with paired VAB and surgical pathology data were evaluated (**Figure 1**). Factors predictive of PRP were analyzed. In total, five of 116 (4.3%) cases had multicentric or bilateral breast cancers, three had VAB only of one lesion, and one had VAB from two different multicentric nodules. All cancers were reported separately in the data.

### Baseline characteristics

Baseline characteristics, imaging findings, and VAB details of all 116 cases are outlined in **Table 1**. Diagnostic ordinary VAB was carried out in 51 of 116 (44%) and EVAB in 65 of 116 (56%). On VAB analysis, 26 cases (22.4%) with invasive disease only, 47 cases (40.5%) with invasive/microinvasive disease with DCIS, and 43 cases (37.1%) with DCIS only.

**Table 1:** Baseline characteristics, imaging findings, and VAB details of all 116 patients evaluated.

Patient characteristics	Total number = 116
Mean age in years (range)	55.66 ± 12.27 (20–91)
Mammograms/ultrasound findings Mass Calcification Mass with calcification	49 (42.2%) 36 (31.0%) 31 (26.7%)
Tumor size on imaging (mm) Mean ± SD (range)	11.67 ± 10.59 (4–88)
Multifocal Yes No	15 (12.9%) 101 (87.0%)
Multicentric/bilateral disease Yes No	5 (4.3%) 111 (95.7%)
Procedure Ordinary VAB EVAB	51 (44%) 65 (56%)
Image-guided approach US Stereotactic	76 (65.5%) 40 (34.5%)
Tumor size measured on VAB pathology (mm) Mean ± SD (range)	5.29 ± 2.89 (1–25)
VAB pathology diagnosis Invasive cancer Invasive cancer with DCIS DCIS DCIS with microinvasion	26 (22.4%) 36 (31.0%) 43 (37.0%) 11 (9.5%)

### Surgical management

Surgical details are summarized in **Table 2**. Breast-conserving surgery was performed in 89 cases (76.7%) and mastectomy in 26 cases(22.4%). For the 32 patients who did not undergo axillary surgery, 27 had pure DCIS. There were five invasive cancers that did not undergo axillary surgery: four elderly patients with immunohistochemistry-like luminal subtype invasive breast cancers (surgical team decision) and one 53-year-old patient with invasive local recurrence and previous axillary dissection. For 11 cases of pure DCIS undergoing SNB, six were mastectomies and five were breast-conserving procedures, in which three cases were suspicious for microinvasion, one was clinically suspicious for invasion, and one was extensive high-grade DCIS with comedonecrosis.

**Table 2:** Details of definitive pathology after VAB and surgery in 116 patients.

Final surgery following VAB Breast-conserving surgery Mastectomy Unknown	89 (76.7%) 26 (22.4%) 1 (0.9%)
Measure of residual IC on surgery (mm)	Mean ± SD = 2.95 ± 5.48 (range: 0–25)
Measure of residual DCIS on surgery (mm)	Mean ± SD = 7.44 ± 11.21 (range: 0–65)
Final tumor invasive size* (mm)	Mean ± SD = 8.38 ± 6.23 (range: 0.8–25)
Final tumor *in situ* size^*^ (mm)	Mean ± SD = 12.61 ± 11.63 (range: 2–65)
Residual disease findings at surgery: No residual tumor Minimal invasive residual disease Minimal *in situ* residual disease Minimal invasive and *in situ* residual disease Gross invasive residual disease Gross *in situ* residual disease Gross invasive and *in situ* residual disease DCIS with microinvasion residual disease	29 (25%) 3 (2.6%) 9 (7.7%) 6 (5.2%) 13 (11.2%) 29 (25%) 19 (16.4%) 8 (6.9%)
Nuclear grade 1 2 3	12 (10.3%) 56 (48.3%) 48 (41.4%)
Histologic grade** 1 2 3 Unknown	18 (25.7%) 34 (48.6%) 16 (22.9%) 2 (2.8%)
Presence of DCIS with comedonecrosis Yes No Unknown	57 (49.1%) 58 (50%) 1 (0.9%)
Nodal status N0 N1-2 No axillary surgery	75 (64.7%) 9 (7.7%) 32 (27.6%)
Estimated subtypes of invasive disease based on immunohistochemistry evaluation Luminal A Luminal B Luminal-HER HER2 enriched Triple negative Pure DCIS at VAB and surgery	32 (27.6%) 25 (21.5%) 7 (6.0%) 6 (5.2%) 8 (6.9%) 38 (32.8%)

*Final tumor size is referred as the largest pathological tumor size measured either on the VAB/EVAB or the surgical specimen, following the TNM stage system (17).

**Pure DCIS (38 cases) and DCIS with microinvasion (eight cases) were excluded from this analysis.

On final pathological analysis (VAB and surgical specimen), 78 of 116 (67.2%) had invasive disease with or without DCIS and 38 of 116 (32.8%) with pure DCIS only.

***Characteristics of tumors potentially resected percutaneously***

In total, 25% of lesions were completely resected by VAB, with a further 15.5% having MRD only. Thus, 40.5% of tumors were classified as PRP. **Table 3** summarizes the qualitative characteristics and **Table 4** the quantitative characteristics of patients grouped according to completeness of excision. 

**Table 3:** Continuous characteristics of all groups and univariate analysis.

	PRP			NPR			*P*-value*
	Complete resected by VAB	Minimal residual disease on surgical pathology	Total	Gross residual disease	Upgrade	Total	
Number of patients	29 (25%)	18 (15.5%)	47 (40.5%)	64 (55.2%)	5 (4.3%)	69 (59.5%)	
Final diagnosis: IC IC+ DCIS DCIS DCIS with microinvasion	12 9 8	0 15 2 1	12 (25.5%) 24 (51.1%) 10 (21.3%) 1 (2.1%)	6 24 28 6	0 4 0 1	6 (8.7%) 28 (40.6%) 28 (40.6%) 7 (10.1%)	< 0.001
Age Mean ± SD (range)	59.31 ± 12.99 (31–91)	59.22 ± 13.39 (33–79)	59.27 ± 12.9 (31–91)	52.80 ± 11.32 (20–76)	58.40 ± 8.79 (44–66)	53.20 ± 11.19 (20–76)	0.008
Tumor size on image (mm), Mean ± SD (range)	8.03 ± 4.02 (4–25)	9.28 ± 3.29 (5–18)	8.50 ± 3.77 (4–25)	15.49 ± 14.47 (4–88)	9.33 ± 4.04 (7–14)	15.00 ± 14.00 (4–88)	0.008
Final tumor invasive size** (mm) Mean ± SD (range)	4.99 ± 2.17 (2–11)	5.31 ± 2.04 (1–8)	5.11 ± 2.10 (1–11)	12.10 ± 9.93 (1–25)	4.76 ± 4.48 (0.8–12)	10.66 ± 7.10 (0.80–25)	<0.001
Final tumor *in situ* size** (mm) Mean ± SD (range)	3.88 ± 1.25 (2–6)	2.25 ± 0.35 (2–2.5)	3.55 ± 1.30 (2–6)	15.96 ± 11.98 (5–65)		15.96 ± 11.98 (5–65)	<0.001
Ki67 (%)	13.50 ± 14.99 (2–80)	21.56 ± 21.17 (3–80)	16.65 ± 17.88 (2–80)	25.80 ± 19.29 (2–90)	18.00 ± 21.68 (5–55)	25.46 ± 19.21 (2–90)	0.016

*t-test.

**Final tumor size is referred as the largest pathological tumor size measured either on the VAB/EVAB or the surgical specimen, following the TNM stage system (17).

**Table 4:** Categorical characteristics of all groups and univariate analysis.

	PRP			NPR			*P*-value
	Complete resected by VAB *N* = 29	Minimal residual disease on surgical pathology *n* = 18	Total *N* = 47	Gross residual disease *n* = 64	Upgrade *N* = 5	Total *N* = 69	
VAB pathology diagnosis: IC (IDC/ILC) DCIS IC+DCIS DCIS w/microinvasion	12 (10 + 2) 8 9 0	4 2 11 1	16 (34%) 10 (21.3%) 20 (42.6%) 1 (2.1%)	10 28 16 10	0 5 0 0	10 (14.5%) 33 (47.8%) 16 (23.2%) 10 (14.5%)	0.001
Presence of DCIS with comedo necrosis: Yes No Unknown	4 24 1	5 13	9 (19.1%) 37 (78.7%) 1 (2.2%)	45 19	3 2	48 (69.6%) 21 (30.4%)	< 0.001
Nuclear grade: 1 2 3	7 20 2	2 12 4	9 (19.1%) 32 (68.1%) 6 (12.8%)	2 24 38	1 0 4	3 (4.3%) 24 (34.8%) 42 (60.9%)	< 0.001
Histologic grade: 1 2 3 Unknown	10 8 2 1	2 10 3	12 (33.3%) 18 (50.0%) 5 (13.9%) 1 (2.8%)	5 14 10 1	1 2 1	6 (17.7%) 16 (47,0%) 11 (32,4%) 1 (2,9%)	0.083
Estimated subtypes of IC based on immunohistochemistry evaluation: Luminal A Luminal B Luminal-HER Her-2 enriched Triple negative	13 5 1 1 1	8 5 1 0 2	21 (56.8%) 10 (27.0%) 2 (5.4%) 1 (2.7%) 3 (8.1%)	8 14 5 4 5	3 1 0 1 0	11 (26.8%) 15 (36.6%) 5 (12.2%) 5 (12.2%) 5 (12.2%)	0.074
Luminal Her negative tumors (78 patients evaluated): Yes No	18 3	13 3	31 (83.8%) 6 (16.2%)	22 14	4 1	26 (63.4%) 15 (36.6%)	0.043
Her-2 (113 patients evaluated): Negative (0 or 1+) Equivocal (2+) Positive (3+)	26 0 3	16 0 2	42 (89.4%) 0 5 (10.6%)	38 2 21	4 0 1	42 (63.6%) 2 (3%) 22 (33.3%)	0.004
Ki-67 (112 patients evaluated): < 14 ≥ 14	19 9	9 9	28 (60.9%) 18 (39.1%)	18 43	3 2	21 (31.8%) 45 (68.2%)	0.004
Image-guided approach: US ST	24 5	14 4	38 (80.9%) 9 (19.1%)	36 28	2 3	38 (55.1%) 31 (44.9%)	0.004
Procedure Ordinary VAB EVAB	7 22	4 14	11 (23.4%) 36 (76.6%)	38 26	2 3	40 (58%) 29 (42%)	< 0.001
Image finding Mass Mass w/calcifications Calcifications	19 6 4	10 5 3	29 (61.7%) 11 (23.4%) 7 (14.9%)	20 17 27	0 3 2	20 (29%) 20 (29%) 29 (42%)	0.001
Multifocal Yes No	2 27	1 17	3 (6.4%) 44 (93.6%)	12 52	0 5	12 (17.4%) 57 (82.6%)	0.083
Multicentric Yes No	1 28	2 16	3 (6.4%) 44 (93.6%)	2 62	0 5	2 (2.9%) 67 (97.1%)	0.394
Recurrence Yes No	3 26	1 17	4 (8.5%) 43 (91.5%)	3 61	0 5	3 (4.3%) 66 (95.7%)	0.439
Nodal status 0+ N+ No axillary surgery	19 3 7	14 1 3	33 (70.2%) 4 (8.5%) 10 (21.3%)	39 5 20	4 0 1	43 (62.3%) 5 (7.3%) 21 (30.4%)	0.915
Final tumor size*– 113 patients evaluated: ≤ 10 mm > 10 mm	27 1	18 0	45 (97.8%) 1 (2.2%)	33 29	4 1	37 (55.2%) 30 (44.8%)	< 0.001
Final tumor size*– 113 patients evaluated: ≤ 5 mm > 5 mm	18 10	8 10	26 (56.5%) 20 (43.5%)	17 45	3 2	20 (29.9%) 47 (70.1%)	0.006

Chi-square test.

IDC, invasive ductal cancers; ILC, invasive lobular cancers.

*Final tumor size is referred as the largest pathological tumor size measured either on the VAB/EVAB or the surgical specimen, following the TNM stage system (17).

**Figure 2:**
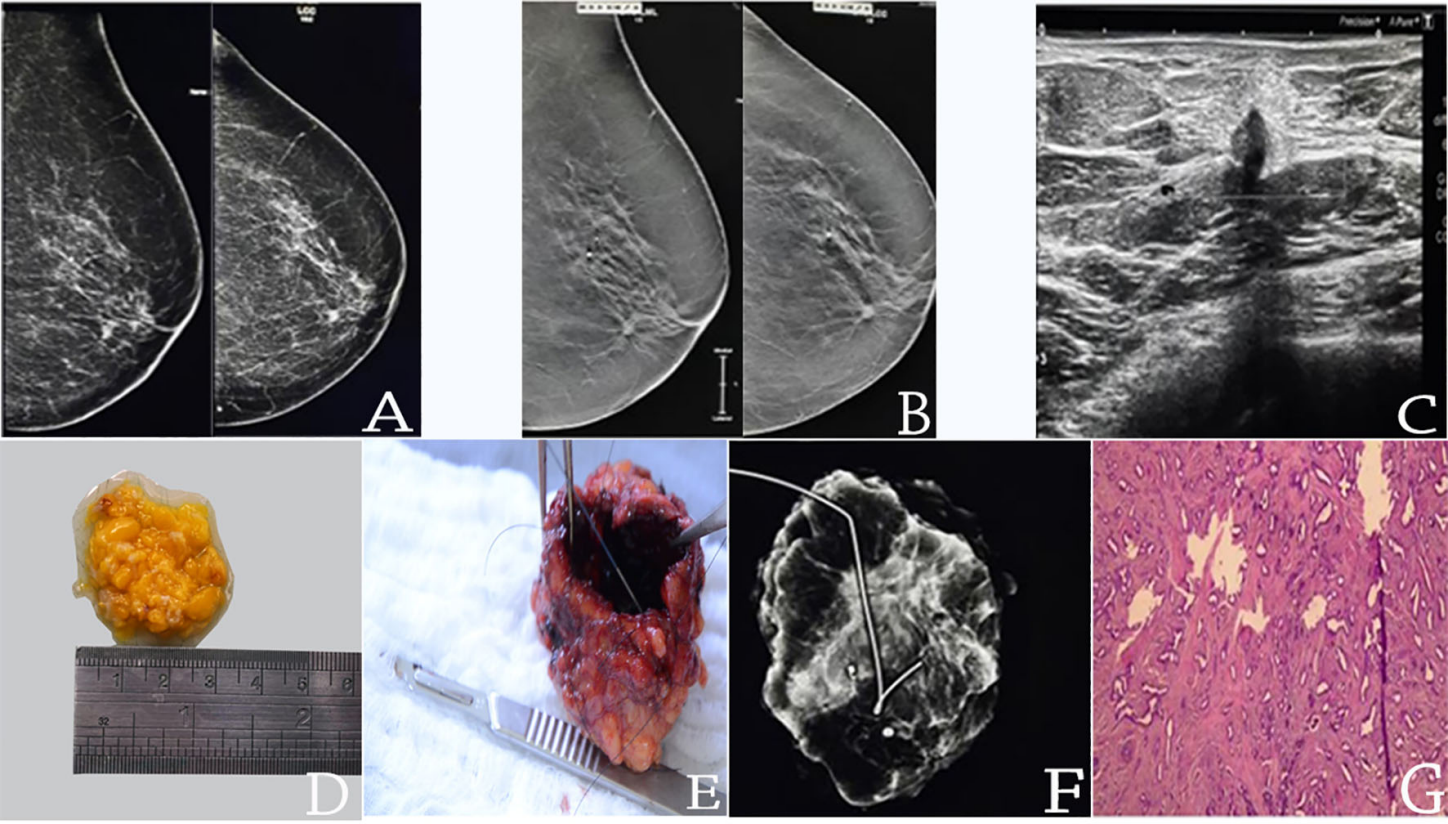
pT1b(8mm)pN0(0/4)sn luminal B invasive breast cancer completed resected by EVAB (36-core samples 10G needle); **(A)** MLO/CC mammograms; **(B)** MLO/CC tomossintesis slices; **(C)** US mass; **(D)** EVAB specimen; **(E)** Surgical specimen after resection; **(F)** radiography of the surgical specimen with the marker on EVAB site **(G)** HE histological slide of EVAB sample, with invasive tubular carcinoma with desmoplastic reaction.

PRP patients generally were older (mean age 59.2 years ± 12.9 SD in PRP x 53.2 years ± 11.1 SD NPR, p= 0.008), used to present more often with invasive disease ( 78.7% of IC+- DCIS in the PRP x 59.4% of cases of IC +- DCIS in NPR, p<0.001), tumor size of ≤10mm ( 97.8% in PRP x 55.2% in NPR, p<0.001), the presence of a mass lesion ( 85.1% in PRP x 58% in NPR, p=0.001) and an ultrasound guided procedure (80.9% in PRP x 55.1% in NPR, p=0.004). **Figure 2** illustrates a complete resected case.

When invasive disease alone was considered, there was no association between immunohistochemistry-like tumor and completeness of excision with VAB (although numbers in each subtype were small). Subgroup analysis of the completely excised invasive tumors demonstrated an overall small mean tumor size (7.5 mm), all presented as either a mass or a mass with calcification, and none presented as pure mammographic calcification. In total, 19 of these cases underwent SLNB—16 were node negative and three were node positive cases—all were pT1a/b tumors, and two were immunohistochemistry-like luminal A subtype and one luminal B.

### Predictors of tumors potentially resected percutaneously

There were 47 (40.5%) tumors which were PRP, of which 10 (21.3%) were DCIS and 37 (78.7%) were invasive disease (12 pure IC, 24 IC + DCIS and 1 DCIS with microinvasion). When PRP was considered in univariate analysis, 17 factors were statistically associated considering *p* ≤ 0.05 (**Tables 3**, **4**).

In multivariate analysis, only four factors remained associated with PRP as shown in **Table 5**: A EVAB procedure (*p* = 0.008, OR: 4.4, 95% CI), low/intermediate nuclear grade (*p* < 0.001, OR: 12.5, 95% CI), final T ≤ 10 mm (*p* < 0.001, OR: 50.1, 95% CI), and final T ≤ 5 mm (*p* < 0.004, OR: 4,2, 95% CI).

**Table 5:** Multivariate analysis for tumors potentially resected and treated percutaneously.

				95% confidence Interval for OR	
	B	*P*-value	OR	Inferior	Superior
**EVAB**	1.474	0.008	4.365	1.465	13.011
**Low/intermediate nuclear grade**	2.528	< 0.001	12.523	3.962	39.585
**Final T ≤ 10 mm**	3.915	< 0.001	50.162	5.792	434.406
**Constante**	−1.702	< 0.001	0.182		
				95% confidence Interval for OR	
	B	*P*-value	OR	Inferior	Superior
**EVAB**	1.499	0.003	4.478	1.657	12.096
**Low/intermediate nuclear grade**	2.309	< 0.001	12.068	3.426	29.590
**Final T ≤ 5mm**	1.445	0.004	4.241	1.574	11.429
**Constante**	−1.854	< 0.001	0.157		

Hosmer-Lemeshow test = 0.435; log likelihood = 106.95; pseudo R = 0.444.

Hosmer-Lemeshow Test = 0.450; log likelihood = 88.38; pseudo R = 0.582.

## Discussion

VAB is a convenient outpatient percutaneous procedures carried out under local anesthesia, which are well tolerated. They can be performed with vacuum biopsy devices, which are used globally for diagnostic purposes. This retrospective series of unselected cases submitted to diagnostic VAB showed that 25% of tumors were completely resected, with a further 15.5% of cases having MRD only. However, a further 55.2% of cases had gross residual disease following VAB, with 4.3% showing an upgrade from DCIS to invasive disease at surgery. This data suggests that with careful selection of patients likely to have very small low risk disease, or indolent disease unlikely to progress, it may be possible to completely resect breast cancer with percutaneous minimally invasive techniques.

In this series, in the group of PRP, only 23% of cases were ordinary VAB and 76% were EVAB. In the other group of tumors NPR, 58% of cases were submitted to ordinary VAB and only 42% to EVAB, showing that for potential CR purpose a EVAB procedure should be recommended. Most of the cases with CR were tumors smaller than 10 mm (96.4%), of low/intermediate nuclear grade (93.1%), IC (72.4%), presenting with a mass lesion either with or without associated mammographic calcifications (86.2%). In addition, in the PRP group 97.8% of tumors were smaller than 10 mm and 87.2% of tumors were nuclear grade 1 or 2. For invasive breast cancers, the data shows that it is possible to complete resect percutaneously invasive ductal (19 of 21, 90.5%) and invasive lobular (2 of 21, 9.5%) carcinomas of all immunohistochemical subtypes smaller than 11 mm in radiological evaluation, although the number of patients in each subtype is relatively small.

Classically, the TNM stage system is used for breast cancer staging (17). Pathological tumor size is defined by the largest tumor size or the largest foci where there is more than one. Following this concept, the largest pathological tumor size either on VAB/EVAB or surgical specimen was used to pathological stage.

In those patients with invasive tumors completely resected by VAB, three (14%) were found to have nodal disease at SLNB, despite negative radiological staging at diagnosis—two luminal A tumors and one luminal B tumor according to immunohistochemical subtyping. All were pT1a/b tumors. Given the increasing use of molecular profiling in identifying patients at genomically low risk despite nodal disease (18), the question remains whether SLNB adds valuable prognostic information in the setting of minimally invasive local treatment or whether SLNB can be safely omitted in this setting.

VAB was a less effective approach for percutaneous resection of DCIS. The largest DCIS completed resected by VAB was 6 mm. DCIS represented just 8 of 29 (27.6%) of all tumors CR and 10 of 47 (21.3%) of PRP compared with 21 of 29 (72.4%) and 37 of 47 (78.7%), respectively, for IC (p 0.001). Additionally, in this series, there were five cases of DCIS, which were upgraded from DCIS to IC following surgery. In our series, lesions presenting with calcifications alone in the absence of an associated mass were much less likely to be completely excised.

Analysis of extent of residual disease following surgical excision allowed the evaluation of VAB for potential minimally invasive resection of breast cancer. In this case series, the procedure was performed with diagnostic rather than therapeutic intent. MRD was defined as ≤ 3 mm of IC/DCIS residual disease in the final excision surgical specimen. This definition was used, because it could therefore be hypothesized that those cases could have been potentially resected in a procedure carried out with therapeutic intent (one or two more core samples); furthermore, focally positive margin is defined as cancer invading for less than 4 mm in length from the inked margin (19), and omitting re-excision for focally positive margins after breast-conserving surgery does not impair disease-free and overall survival (20) or the local regional recurrence rate (21, 22) in the context of appropriate adjuvant radiotherapy and endocrine therapy. Although close margins are defined as tumor less than 2 mm width from the inked margin (23), the rate of residual disease on close and focally positive margins is similar (22, 23).

Screening mammography has been associated with reduced mortality from breast cancer in women 40–70 years of age, with absolute risk reduction of 0.809 (0.742–0.833 CI) (24). Benchmarks reported by the Breast Cancer Surveillance Consortium (BCSC) for mammography screening are median size of IC of 14 mm, 77.3% of node negative cancers, 52.6% of minimal cancers (less than 1 cm invasive cancers or *in situ*), and 74.8% of stage 0 and 1 cancers (25). Most of cancers detected on screening programs are small node negative cancers potentially eligible for percutaneous treatment.

Overdiagnosis in mammographic screening programs remains a significant problem, with reported rates ranging from 11%–22% (24–28). This has largely been attributed to a substantial increase in the detection of small tumors, often with favorable biological features, without any corresponding reduction in the incidence of larger tumors, nor indeed of metastatic disease (26). Local therapy remains an integral part of the treatment of such cancers; however, it is necessary to tailor treatment approaches to the needs and preferences of the individual, minimizing treatment morbidity as far as possible while preserving oncological safety (15). Thus, there is increasing interest using minimally invasive approaches for the local therapy of breast cancer, in part to address the issues around overdiagnosis and consequent overtreatment.

In this context, several percutaneous ablative techniques have been described, with high-technical efficacy rates (> 80%), low complication rates, and acceptable local recurrence rates (10, 29). More recently, interim analysis of the ICE3 prospective, single-arm trial evaluating cryoablation for low-intermediate grade, biologically favorable tumors <15 mm in women aged 60 years or older demonstrated an ipsilateral breast tumor recurrence rate of 2% after a mean follow-up of 35 months (30). For 194 patients, who received successful cryoablation, the mean age was 75 years (range: 55–94 years). The mean tumor length was 8.1 mm (range: 8 mm–14.9 mm), and the mean tumor width was 7.4 mm (range: 2.8 mm–14 mm). Therefore, it appears that ablation techniques may be useful in treating early stage breast cancer, although there is a paucity of high-quality, randomized evidence comparing them with surgery to support adoption into clinical practice. However, ablative techniques by their nature disrupt the tumor and do not provide tissue for evaluation, which is required for histopathological assessment and increasingly for molecular profiling to guide selection of systemic therapies.

Tumor size on image for PRP by VAB was 8.50 mm ± 3.77 (4–25). VAB differs from cryoablation, as cryoablation is an ablative technic, whereas VAB is based on resection allowing pathological evaluation of the specimen.

Another percutaneous excisional technique, which allows histopathological evaluation of tumor margins, is the Breast Lesion Excision System (BLES). The IPEX trial used BLES, which excises the lesion whole rather than piecemeal (31). The IPEX trial reported 124 cases of DCIS or IC, and following excision, 101 (81%) had clear histologic margins [average lesion size was 11 mm for both invasive cancers (4 mm–20 mm) and DCIS (1.5 mm–20 mm)]. However, the BLES device has now been withdrawn from the market, and has not been a widely available technique, unlike VAB technologies that are in common use across the globe.

Inevitably, there are some limitations to a retrospective single-center case series of this nature. Patients were undergoing a diagnostic procedure rather than a therapeutic procedure. Patient numbers are relatively small, particularly in respect of the immunohistochemical subtype analysis, which means that it is difficult to draw definitive conclusions about the ability of VAB to fully excise different subtypes of breast cancer. However, this study adds to the weight of data supporting the potential use of VAB for the treatment of small, biologically favorable screen-detected invasive breast cancers. The ideal evaluation of this technique would require a large, multicenter randomized study such as the UK SMALL trial (10). SMALL is a prospective, multicenter, randomized phase III trial of minimally invasive VAE procedure *versus* surgery in patients with small (≤ 15 mm), biologically favorable screen-detected breast cancer conducted in NHS, United Kingdom, which is recruiting patients. The aim of the trial is to generate high-quality, practice-changing clinical evidence to support the safe de-escalation of surgical treatment within the context of standard adjuvant radiotherapy and endocrine therapy in selected patients and will evaluate both the requirement for re-excision after VAE and long-term local recurrence rates.

## Conclusions

This data provides further evidence that small, low/intermediate nuclear grade pT1a/b breast tumors presenting as a mammographic/ultrasound mass lesion can be potentially completely resected percutaneously by VAE. Prospective trials supporting minimally invasive techniques are required to support evidence-based change in surgical practice.

## Data availability statement

The original contributions presented in the study are included in the article/supplementary materials. Further inquiries can be directed to the corresponding author.

## Ethics statement

The studies involving humans were approved by Ethical Committee of Santa Casa de Belo Horizonte by the number 25761019.8.0000.5138. The studies were conducted in accordance with the local legislation and institutional requirements. The participants provided their written informed consent to participate in this study.

## Author contributions

All authors contributed to many steps of the research, including study concepts, study design, data acquisition, data analysis and interpretation, statistical analysis, manuscript preparation, manuscript editing and/or manuscript revision. All authors contributed to the article and approved the submitted version.
